# Human Papillomavirus (HPV) Infection: Molecular Epidemiology, Genotyping, Seroprevalence and Associated Risk Factors among Arab Women in Qatar

**DOI:** 10.1371/journal.pone.0169197

**Published:** 2017-01-03

**Authors:** Asha A. Elmi, Devendra Bansal, Anushree Acharya, Sini Skariah, Soha R. Dargham, Laith J. Abu-Raddad, Nady Mohamed-Nady, Paul Amuna, Asma A. J. Al-Thani, Ali A. Sultan

**Affiliations:** 1 Department of Microbiology and Immunology, Weill Cornell Medicine—Qatar, Cornell University, Qatar Foundation—Education City, Doha, Qatar; 2 Infectious Disease Epidemiology Group, Weill Cornell Medicine—Qatar, Cornell University, Qatar Foundation—Education City, Doha, Qatar; 3 Department of Obstetrics & Gynecology, Weill Cornell Medicine—Qatar, Cornell University, Qatar Foundation—Education City, Doha, Qatar; 4 Primary Health Care Corporation, Doha, Qatar; 5 Health Sciences Department, Biomedical Sciences Program, University of Qatar, Doha, Qatar; University of Louisville School of Medicine, UNITED STATES

## Abstract

Human Papillomavirus (HPV) infections are known to cause cervical cancer worldwide, however, limited information is currently available on prevalence, types distribution and risk factors for HPV infection in the Arab countries. We conducted a cross-sectional observational study exclusively of women of Arabic origin residing in Qatar (n = 406) who were selected from the Women’s Hospital at Hamad Medical Corporation (HMC) and Health Centers of the Primary Health Care Corporation in Doha, Qatar over the period March 2013 to August 2014. Socio-demographic, behavioral and clinical data were collected. Four hundred and six cervical smears and 292 blood samples were included in the study. HPV typing was done using HPV type-specific primers-based real-time PCR, and Sanger sequencing. HPV-IgG and IgM were quantified using ELISA assays. The prevalence of HPV infection amongst Qatari and non-Qatari Arab women were 9.8% and 6.1%, respectively and 7.6% and 16.7% in women with normal and abnormal cytology, respectively. HPV 81 was the most commonly found genotype in women with normal cytology (34.5%), whereas HPV 81, 16 and 59 in women with abnormal cytology (25.0% each). All the HPV DNA positive women were seronegative and HPV-IgG prevalence was higher in Qatari women than in non-Qatari Arab women. None of the studied factors had any significant association with HPV-DNA positivity or HPV-IgG seropositivity. The overall identified HPV DNA prevalence and HPV seroprevalence among Arab women in Qatar were on the low side compared to global levels.

## Introduction

Human Papillomavirus (HPV) is one of the most prevalent sexually transmitted infections worldwide, and molecular studies have implicated infection with specific HPV high-risk (HR) genotypes as etiological agents of cervical cancer [1–3]. Several factors influence and contribute to the development of cervical cancer. There are over 200 unique HPV genotypes that have been confirmed, of which 30 to 40 are categorized on the basis of their oncogenic potential as HR and low-risk (LR) leading to cervical neoplasia and mild dysplasia, respectively [4]. According to the World Health Organization (WHO), cervical cancer, although can be preventable, is responsible for over 270,000 death annually and is the second commonest type of cancer among women worldwide [5]. It is the fifth most diagnosed cancer amongst women in the State of Qatar [6]. Knowledge of HPV status improves cervical cancer screening and triage for women with mild or borderline cervical smears and HPV vaccination program. However, the guidelines for cervical cancer screening in Qatar are currently under review. As of today, most of the screenig for cervical cancer is opportunistic, in which either the physician is offering or the patient is requesting the test and the frequency is offered every 3 years for low risk patients. HPV vaccines are licensed in Qatar, but currently there is no vaccination program in the country. The vaccine is available however for those who are interested in the vaccine. HPV testing is also not routinely practiced, but it is often performed when a Pap smear result is abnormal or the patient request the test.

Though the incidence of cervical cancer in the Extended Middle East and North Africa (EMENA) shows lower rates compared to the rest of the world [2, 7], the burden of HPV infection still warrants public health interventions. In addition, the age specific HPV prevalence has varied widely across different population and showed two peaks of HPV positivity in younger and older women[8,9]. Among the general population of Arab women with normal or abnormal cytology residing in Qatar, we recently estimated an HPV prevalence rate of 6.1% [10]. We also identified the presence of a varied genotypic profile of HPV with a high prevalence of low-risk HPV genotype 81 [10]. However, HPV DNA testing cannot differentiate between current and previous infection and does not reflect the lifetime risk of HPV infection. Moreover, despite much progress, risk factors influencing the epidemiology of HPV infection are not yet fully understood [11].

During HPV infection, both humoral and cellular immune responses are induced, and antibody production against HPV is important for preventing the spread of infection and re-infection [12,13]. Additionally, it has been shown that the cell-mediated immune response cleared the majority of HPV infections within 1–2 year’s exposure [14]. Despite the fact that a number of seroprevalence studies have been conducted in resource-rich countries and some in resource-poor countries [15–18], no such data appears to be available from the Arab world.

Qatar, a country located in the Arabian Peninsula has in recent years experienced rapid economic growth and globalization resulting in a large influx of foreign expatriates from Western, other Middle Eastern, African and Asian countries. The total population of women of child-bearing age was reported as 447,298; of which 382,067 represent Arabic women (Qatari and non-Qatari) according to the Ministry of Development Planning and Statistics, Qatar [6]. The economic and demographic transition and the resulting dynamic socio-economic and socio-cultural environment, may affect the social and sexual behaviours in the country. However, how these changes affected the behavioral risk factors and impacts on the prevalence of HPV infection among Arab women is yet to be determined.

Therefore, in the present study, we extended our analysis to identify the potential risk factors for HPV acquisition along with HPV seroprevalence among Arab women, with normal or abnormal cytology, residing in the State of Qatar. The association of risk factors with HPV-DNA positivity and HPV-IgG seropositivity was also analyzed. In addition, HPV prevalence and genotypes were correlated with cytology results. It is hoped that the outcomes of this study will inform the evaluation of the relevance of HPV vaccination and HPV screening in the State of Qatar and the development of policies and guidelines for cervical cancer prevention.

## Materials and Methods

### Study population and sample collections

This cross-sectional study was conducted according to the principles expressed in the Declaration of Helsinki. This study was reviewed and approved by Research Committee of Hamad Medical Corporation, Weill Cornell Medicine-Qatar and Primary Health Care Corporation (PHCC), Doha, Qatar. All subjects and/or significant others provided written informed consent prior to their participation.

The sample was convenient sample of 406 Arab women, nationals of any of the 22 countries in the League of Arab States, attending the Women’s Hospital at HMC and PHCC. A sample size of 430 was calculated to estimate an HPV prevalence of 4% (precision rate = 2%; a reasonable precision given the expected low HPV prevalence) and a significance level of 5% (type I error α = 0.05). The refusal rate was 4% and mainly to time availability of the patients. Cervical samples were collected from all the subjects (406), of which only 292 individuals provided matched blood samples. Subjects refused to give a blood sample for several reasons such as they do not want to give blood, do not want to spend more time in the clinic, and also because of reluctance to undergo an extra medical procedure. Cervical samples were collected in ThinPrep vials (BD SurePath™) for Pap smear assay and molecular HPV typing. ThinPrep cytological smears were screened and evaluated at HMC and reported according to the Bethesda system for reporting of cervical cytology [19]. Whole blood samples were collected in plane tubes and sera was obtained by centrifuging the blood samples at 1902 g for 5 minutes and stored at -80°C for further use. Inclusion criteria for the study comprised married or previously married, non-pregnant women. Never married women were excluded from participating in this study since their participation was deemed culturally unacceptable. In addition, women with known diagnosis of cervical cancer and immunocompromised patients were also excluded from the study.

### HPV risk factor survey

All subjects were interviewed using a structured questionnaire to collect socio-demographic and behavioral information on possible risk factors for HPV infection such as marital status, education, household income, smoking, contraceptive practices, knowledge about HPV, HPV vaccine and cervical cancer. The questionnaire was prepared on the basis of a previous study conducted in Qatar (S1 Questionnaire) [20]. Other data from medical records include age, nationality, clinical history, and cytological diagnosis were collected for each subject.

### DNA isolation and HPV testing by real time PCR and sequencing

Viral DNA was extracted from cervical samples using QIAamp MinElute virus spin kit (Qiagen, CA, USA) as previously described [10]. To detect HPV-DNA, real-time polymerase chain reaction (RT-PCR) assay was carried out in ABI 7500 (Applied Biosystems, CA, USA) and PCR reaction mixture and amplification conditions for GP5+/GP6+ primers (HPV, L1 consensus primers) and PCO3/PCO4 primers (human β globin gene) have been previously described [10]. A positive control (cloned HPV-DNA) and a negative control (nuclease free water) were included in each amplification reaction. HPV DNA positivity among clinical samples was detected as previously described [10].

Real Time PCR-based kits (Sacace Biotechnology, Como, Italy) and Sanger method (Genewiz, NJ, USA) were used to identify the genotype(s) in the samples which tested positive for HPV DNA, as described previously [10, 21].

### Detection of HPV IgG and IgM antibodies by Enzyme Linked Immunosorbent Assay (ELISA)

Human Papillomavirus IgG (HPV-IgG) and IgM (HPV-IgM) were quantified by double-antibody sandwich ELISA kits (Novateinbio, Cambridge, USA) according to the manufacturer's instructions. Briefly, 50 μl serum samples and 100 μl HRP conjugated antibody was added to pre coated 96 flat-bottomed plates and incubated for 1h at 37°C. Following incubation, unbound substances were removed by washing solution (5X) and finally, HRP substrate (50 μl of each Chromogen A and Chromogen B solution) was added and plate was incubated for 15 min at 37°C. After appearance of yellow color in negative wells, the reaction was stopped with 50 μl stop solution. The optical density (OD) was measured at 450 nm using an ELISA plate reader and results were analyzed. The cutoff value was calculated using the manufacturer’s instructions. The average OD of the negative control wells plus 0.15 was taken as the cutoff critical value. A negative HPV result was interpreted as sample OD less than the calculated cutoff value, and samples with an OD greater than the calculated cutoff value were reported as positive for HPV IgG.

### Statistical analysis

Statistical analyses were performed using SPSS (IBM SPSS version 23.0). Sample characteristics including age, nationality, marital status, education level, household monthly income, smoking habits, current method of contraception, knowledge about HPV, HPV vaccine and related cervical cancer, cytology results, and clinical findings were briefed using frequency distributions. Crude association between HPV positivity and each of the previously mentioned characteristics was assessed using the chi-squared test. Significance level was considered at α = 0.05 and unadjusted odds ratios (OR) were reported with their corresponding 95% confidence intervals (CI).

## Results

### Demographic and clinical profiles of subjects

All enrolled Arab women were categorized into 5 broad age groups (21–30, 31–40, 41–50, 51–60, ≥61 years), in which 34.5% of women were aged 41–50 years, 29.8% were aged 31–40 years, 17.5% were aged 51–60 years, 13.3% were aged 21–30 years, and only 4.9% of women were aged 61 years or more. The age range across the sample was 59 years with a mean age of 42.8 years (SD = 10.5 years). Additionally, 55.4% of women (n = 225, 95% CI: 50.7–60.6%) who participated in this study were Qatari nationals, followed by 26.8% (n = 109, 95% CI: 22.7–31.3%) from Fertile Crescent, 10.6% (n = 43, 95% CI: 7.9–13.8%) from Arabian Peninsula, and only 7.1% (n = 29, 95% CI: 4.7–9.9%) from North and East Africa (Table 1). This is reflective of the make-up of the general Arab women population in Qatar. Other demographic and behavioral characteristics are described in Table 1.

**Table 1:** Unadjusted odd ratios (ORs) for HPV DNA positivity and their corresponding 95% confidence intervals (CIs) according to socio-demographic and related characteristics among 406 Arab women in Qatar.

	Total Sample N (%)	HPV patient N (%)	OR	95% CI	p-value
Nationality					
Qatar	225 (55.4)	22 (9.8)	REF		
Arabian Peninsula^$^	43 (10.6)	3 (7.0)	0.692	0.198–2.423	0.565
Fertile Crescent^£^	109 (26.8)	7 (6.4)	0.633	0.262–1.532	0.311
North and East Africa^#^	29 (7.1)	1 (3.4)	0.33	0.043–2.541	0.287
Age					
21–30	54 (13.3)	2 (3.7)	REF		
31–40	121 (29.8)	9 (7.4)	2.089	0.436–10.013	0.357
41–50	140 (34.5)	12 (8.6)	2.437	0.527–11.271	0.254
51–60	71 (17.5)	8 (11.3)	3.302	0.672–16.229	0.242
≥61	20 (4.9)	2 (1.0)	2.889	0.379–22.039	0.306
Marital Status					
Married	352 (88.2)	26 (7.4)	REF		
Separated/Divorced	23 (5.8)	1 (4.3)	0.57	0.074–4.398	0.59
Widowed	24 (6.0)	4 (16.7)	2.508	0.798–7.884	0.116
Education					
No Schooling	43 (10.7)	5 (11.6)	REF		
Elementary—Intermediate	83 (20.7)	9 (10.8)	0.924	0.289–2.952	0.894
Secondary/High school	96 (23.9)	7 (7.3)	0.598	0.178–2.002	0.404
College/University Degree	179 (44.6)	12 (6.7)	0.546	0.182–1.642	0.282
Household Monthly Income					
<5,000 QAR	11 (3.1)	3 (27.3)	REF		
5,000–20,000 QAR	146 (40.9)	11 (7.5)	0.217	0.050–0.938	0.041
> 20,000 QAR	149 (41.7)	13 (8.7)	0.255	0.060–1.080	0.064
Don't know	51 (14.3)	5 (9.8)	0.29	0.058–1.459	0.133
Smoking					
No	371 (96.9)	27 (7.3)	REF		
Yes	12 (3.1)	2 (16.7)	2.548	0.531–12.222	0.242
Current Method of Contraception					
No	287 (71.9)	24 (8.4)	REF		
Yes	112 (28.1)	8 (7.1)	0.843	0.367–1.937	0.687
Birth Control Pills					
No	359 (91.1)	26 (7.2)	REF		
Yes	35 (8.9)	4 (11.4)	1.653	0.542–5.041	0.377
Condom					
No	393 (99.7)	30 (7.6)	REF		
Yes	1 (0.3)	0 (0.0)	a/n	a/n	1-
Intrauterine Device					
No	352 (89.8)	29 (8.2)	REF		
Yes	40 (10.2)	1 (2.5)	0.286	0.038–2.155	0.224
Rhythm Method					
No	388 (98.7)	30 (7.7)	REF		
Yes	5 (1.3)	0 (0.0)	a/n	a/n	1-
Abstinence					
No	390 (99.2)	30 (7.7)	REF		
Yes	3 (0.8)	0 (0.0)	a/n	a/n	1-
Withdrawal					
No	387 (98.5)	28 (7.2)	REF		
Yes	6 (15)	2 (33.3)	6.411	1.125–36.539	0.036
Female Sterilization					
No	373 (94.9)	29 (10.5)	REF		
Yes	20 (5.1)	1 (5.0)	0.624	0.081–4.832	0.652
Knowledge about HPV					
No	349 (88.1)	26 (7.4)	REF		
Yes	47 (11.9)	5 (10.6)	1.479	0.539–4.059	0.447
Knowledge about cervical cancer					
No	29 (7.4)	2 (6.9)	REF		
Yes	363 (92.6)	30 (8.3)	1.216	0.276–5.365	0.796
Knowledge about HPV Vaccine					
No	320 (93.0)	24 (7.5)	REF		
Yes	24 (7.0)	4 (16.7)	2.467	0.780–7.800	0.124
Cytology results					
Normal	382 (94.1)	29 (7.6)	REF		
Abnormal^^^	24 (5.9)	4 (16.7)	2.434	0.780–7.599	0.126
Clinical findings					
Routine smear test	291 (71.7)	23 (7.9)	REF		
Symptomatic results^*^	115 (28.3)	10 (8.6)	1.11	0.511–2.411	0.793

^^^Abnormal cytology includes LGSIL + HGSIL.

^*^Symptomatic results include: cervical erosion, genital warts, lower abdominal pain, menorrhagia, pelvic pain, post coital bleeding, primary infertility, secondary infertility, vaginal bleeding, vaginal spotting and vulva itching.

^$^Arabian Peninsula excluding Qatar (KSA, Kuwait, Bahrain, UAE, Yemen).

^£^Fertile Crescent (Egypt, Iraq, Jordan, Lebanon, Palestine, Syria).

^#^North & East Africa (Morocco, Algeria, Tunisia, Sudan).

Furthermore, data on Table 1 shows that 71.7% (n = 291, 95% CI: 67.5–76.1%) of women underwent routine gynecologic care and only 28.3% (n = 115, 95% CI: 23.9–32.5%) had different clinical symptoms such as vaginal discharge, vaginal bleeding, genital warts, vulva itching, lower abdominal pain, infertility, dyspareunia, polymenorrhagia/menorrhagia, amenorrhea and others. Cytology results show 94.1% (n = 382, 95% CI: 91.6–96.3%) of women with normal cytology with no lesions, and 5.9% (n = 24, 95% CI: 3.7–8.4%) of women with abnormal cytology results, including either low-grade squamous intraepithelial lesion (LGSIL) indicating mild dysplasia; or high-grade squamous intraepithelial lesion (HGSIL) indicating severe intraepithelial neoplasia and atypical cells of undetermined significance (ASCUS).

### Risk factors associated with HPV DNA and type-specific prevalence

A univariate analysis of the association between HPV DNA positivity / type distribution and socio-demographic/behavioural variables are shown in Table 1. The overall HPV prevalence in all Arab women was 8.1% (n = 33, 95% CI: 5.4–11.1%) with a higher prevalence (9.8%, n = 22, 95% CI: 6.2–13.8%) among Qatari women and 6.1% (n = 11, 95% CI: 2.8–9.9%) in non-Qatari women. The results in Table 1 show that there was no statistically significant relationship between HPV infection and nationality (Qatari and Non-Qatari) (*p* = 0.673). Among non-Qatari Arab women, HPV prevalence was highest in women originating from Arabian Peninsula 7.0% (n = 3, 95% CI: 0.0–16.3%), followed by women from Fertile Crescent countries 6.4% (n = 7, 95% CI: 2.8–11.9%). The lowest prevalence of 3.4% (n = 1, 95% CI: 0.0–10.3%) was found in women originating from North and East Africa (Table 1).

HPV prevalence was found to be highest (11.3%) in the age group 51–60 (n = 8, 95% CI: 5.6–18.3%) and lowest (3.7%) in the 21–30 age group (n = 2, 95% CI: 0.0–9.3%) (Table 1). However, no significant difference was found between HPV prevalence vs. age groups. From the cytology results, prevalence of HPV was 7.6% (n = 29, 95% CI: 5.0–10.2%) among women with normal cytology and 16.7% (n = 4, 95% CI: 4.2–33.3%) among those with abnormal cytology. The latter included all the 24 abnormal samples with LGSIL of which four were HPV positive. Of the four samples, three were Qatari women diagnosed with LGSIL (n = 2) and ASCUS (n = 1) and one women was Egyptian diagnosed with LGSIL. Among the Qatari nationals, the most frequent genotypes were LR HPV 11, 90 and 81 along with the HR HPV 16, while only HR HPV 16 was found in the Egyptian national. No HGSIL cases were detected. No significant difference was observed between HPV prevalence and cytology results (OR: 2.43, 95% CI: 0.78–7.60) and HPV prevalence and clinical findings (OR: 1.11, 95% CI: 0.51–2.41) (Table 1).

The risk factors such as marital status (widowed), level of education (no schooling and intermediate), household monthly income (<5000.00 QAR) and smoking tended to have increase of HPV infection risk (16.7%, 11.1%, 27.3% and 16.7%, respectively), however, did not observe any statistical significance. The other risk factors like reproductive factor (contraceptive methods), knowledge about HPV, cervical cancer and HPV vaccine have no influence in HPV infection risk and had no significant association.

Of the total of 406 samples collected in this study population, 33 samples (8.13%) were positive for HPV DNA. These positive samples were further screened to identify the HPV genotypes and were classified based on their oncogenic potential to high-risk (HR) and low-risk (LR) as shown in Table 2. At least one of 8 different genotypes (HR or LR) was detected in the 33 positive samples by HPV genotyping kits and/or DNA based sequencing (Table 2).

**Table 2:** The distribution of HPV types among HPV DNA positive cervical samples among Arab women in Qatar.

	Normal Cytology (N = 382)					Abnormal Cytology (N = 24)				
	Single	Double	Unknown	Total	Percent	Single	Double	Unknown	Total	Percent
**HPV positive**	18	3	8	29	7.59	3	1	0	4	16.67
**High-risk**										
**16**	0	0	-	0	0.00	1	0	-	1	25.00
**33**	0	1	-	1	3.45	0	0	-	0	0.00
**35**	1	1	-	2	6.90	0	0	-	0	0.00
**39**	1	0	-	1	3.45	0	0	-	0	0.00
**59**	0	1	-	1	3.45	0	1	-	1	25.00
**Low-risk**										
**11**	8	1	-	9	31.03	1	0	-	1	25.00
**81**	8	2	-	10	34.48	0	1	-	1	25.00
**90**	0	0	-	0	0.00	1	0	-	1	25.00

Among HPV positive women, taking into account the total frequency of occurrence of each specific genotype occurring either as single/multiple infection (sum of frequency of genotypes belonging to a specific risk category/total number of women (33)), 63.6% (n = 21, 95% CI: 45.5–78.8%) had at least one LR HPV genotype and 18.2% (n = 6, 95% CI: 6.1–33.3%) had at least one HR HPV genotype (Table 2). The most frequent HR genotype among women with normal cytology was HPV 35 (6.9%), while HPV 16 and HPV 59 were the most frequent genotypes among women with abnormal cytology (25%) (Table 2). Furthermore, HPV 81 (34.5%) was the most frequent LR genotype among women with normal cytology; while HPV 11, HPV 81, and HPV 90 had equal distribution among women with abnormal cytology (Table 2). Eight samples (24.2%) remained uncharacterized and all of them were with normal cytology (Table 2). Moreover, single infection and double infections (infection with two different HPV genotypes) were noted. Among women with normal cytology, 18 women (62.1%; 95% CI: 44.8–79.3%) had single infection, and 3 (10.3%; 95% CI: 0.0–24.1%) had double infections (Table 2). However, in women with abnormal cytology, 3 (75.0%; 95% CI: 25.0–100.0%) had single infection, and 1 (25.0%; 95% CI: 0.0–75.0%) had double infections (Table 2).

HPV type-specific was not significantly associated with any of potential risk factors, assessed in the present study, such as marital status, education level, economic status, usage of contraception, smoking habits and knowledge about HPV, HPV vaccine and cervical cancer (Table 1).

### Seroprevalence and associated risk factors in Arab women

We assessed the levels of IgG and IgM in the sera of 292 Arab women residing in the State of Qatar. The seroprevalence of HPV according to different variables is shown in Table 3. The overall HPV-IgG prevalence was 4.5% (n = 13, 95% CI: 2.4–6.8%). The prevalence of HPV-IgG in Qatari women was higher (5.3%, n = 9, 95% CI: 2.3–8.8%) than non-Qatari women 3.3% (n = 4, 95% CI: 0.0–6.6%) (Table 3). Among non-Qatari Arab women, HPV-IgG antibodies were detected higher (9.1%) in women originating from North and East Africa (n = 2, 95% CI: 0.0–22.7%) followed by Fertile Crescent countries 2.8% (n = 2, 95% CI: 0.0–6.9%).

**Table 3:** Unadjusted odd ratios (ORs) for HPV IgG positivity and their corresponding 95% confidence intervals (CIs) according to selected descriptive variables among 292 Arab women in Qatar.

	Total Sample N (%)	IgG Positivity N (%)	OR	95% CI	*p*-value
Nationality					
Qatar	171 (58.6)	9 (5.3)	REF		
Arabian Peninsula^$^	27 (9.2)	0 (0.0)	n/a	n/a	n/a
Fertile Crescent^£^	72 (24.6)	2 (2.8)	0.514	0.108–2.442	0.403
North and East Africa^#^	22 (7.5)	2 (9.1)	1.800	0.363–8.925	0.472
Age					
21–30	38 (13.0)	2 (5.3)	REF		
31–40	81 (27.7)	6 (7.4)	1.440	0.277–7.490	0.665
41–50	99 (33.9)	2 (2.0)	0.371	0.050–2.734	0.331
51–60	58 (19.9)	3 (5.2)	0.982	0.156–6.169	0.984
≥61	16 (5.5)	0 (0.0)	a/n	a/n	0.999
Marital Status					
Married	253 (87.5)	9 (3.6)	REF		
Separated/Divorced	17 (5.9)	3 (17.6)	5.810	1.414–23.874	0.015
Widowed	19 (6.6)	1 (5.3)	1.506	0.181–12.557	0.705
Education					
No Schooling	28 (9.7)	1 (3.6)	REF		
Elementary—Intermediate	64 (22.1)	3 (4.7)	1.328	0.132–13.352	0.81
Secondary/High School	66 (22.6)	2 (3.0)	0.844	0.073–9.702	0.892
College/University Degree	132 (45.2)	7 (5.3)	1.512	0.179–12.802	0.704
Household Monthly Income					
<5,000 QAR	8 (3.1)	0 (0.0)	a/n	a/n	0.999
5,000–20,000 QAR	104 (35.6)	8 (7.7)	REF		
> 20,000 QAR	109 (37.3)	3 (2.8)	0.340	0.088–1.317	0.118
Don't know	41 (15.6)	2 (4.9)	0.615	0.125–3.028	0.550
Knowledge about HPV					
No	246 (86.0)	12 (4.9)	REF		
Yes	40 (14.0)	1 (2.5)	0.5	0.063–3.954	0.511
Knowledge about Cervical Cancer					
No	18 (6.3)	2 (1.1)	REF		
Yes	268 (93.7)	11 (4.1)	0.342	0.070–1.677	0.186
Knowledge about HPV Vaccine					
No	250 (92.6)	10 (4.0)	REF		
Yes	20 (7.4)	2 (1.0)	2.667	0.543–13.102	0.227
Cytology Results					
Normal	274 (93.8)	9 (3.3)	REF		
Abnorma**l**^**^**^	18 (6.2)	4 (2.2)	8.413	2.305–30.704	0.001
Clinical Findings					
Routine Smear Test	217 (74.3)	13 (6.0)	REF		
Symptomatic Results^*^	75 (25.7)	0 (0.0)	n/a	n/a	n/a

^^^Abnormal cytology includes LGSIL + HGSIL.

^*^Symptomatic results include: cervical erosion, genital warts, lower abdominal pain, menorrhagia, pelvic pain, post coital bleeding, primary infertility, secondary infertility, vaginal bleeding, vaginal spotting and vulva itching.

^$^Arabian Peninsula excluding Qatar (KSA, Kuwait, Bahrain, UAE, Yemen).

^£^Fertile Crescent (Egypt, Iraq, Jordan, Lebanon, Palestine, Syria).

^#^North & East Africa (Morocco, Algeria, Tunisia, Sudan).

**Fig 1:**
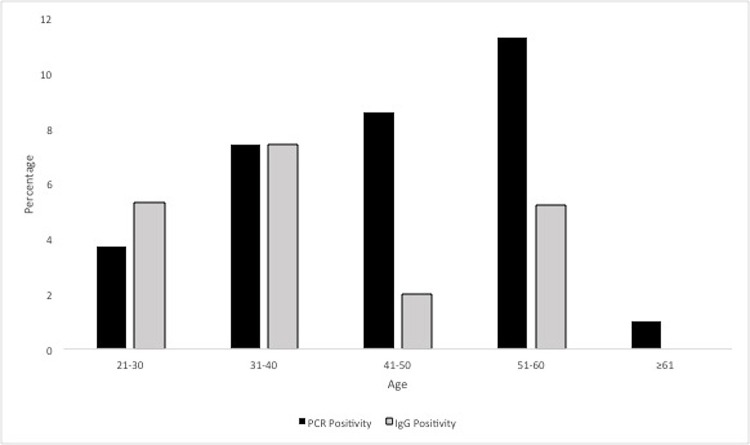
Prevalence of HPV-DNA and HPV-IgG positivity according to age group among Arab women residing in Qatar.

According to age groups, the prevalence of HPV-IgG was highest (7.4%, n = 6, 95% CI: 2.4–13.6%) in the age group 31–40, however, did not differ significantly according to patient age (*P* = 0.615). Additionally, HPV DNA positivity was also correlated with seropositivity by age group, but no significant difference was observed. However, the prevlence of HPV-DNA was higher in the age group 41–60 as compared to the HPV-IgG positive women (Fig 1). Furthermore, the seroprevalence of HPV-IgG was highest among separated/divorced women (17.6%, n = 3) and lowest among married women (3.6%, n = 9), but the difference was not statistically significant (*P* = 0.705). The HPV-IgG seropositivity was slightly higher among women with college/university education, monthly income 5000.00–20,000.00 QAR, knowledge about cervical cancer but not HPV vaccine, no smoking, no use of any contraceptive methods, normal cytology and underwent routine health care, however, had no significant associations with HPV infection. It is noteworthy that all samples were negative for HPV-IgM.

## Discussion

Cervical cancer is a leading cause of cancer death among women worldwide, and approximately 85% of these deaths occur in developing countries [22,23]. It has been shown that genital HPV is the main causal agent for cervical cancer [2]. The high burden of cervical cancer in developing countries reflects the insufficiency and poor capacity of existing screening programs to detect precursor for or early stage of cervical carcinoma [24,25]. Recently, we have shown lower rate of HPV prevalence and heterogeneous distribution of HPV type among Arab women with normal or abnormal cytology [10]. In the present study, we sought to determine the HPV seroprevalence and potential risk factors associated with HPV infection among Arab women living in the State of Qatar.

In this study, HPV DNA prevalence among Arab women in Qatar was found to be slightly higher (8.1%) than we previously report (6.1%) in the large cohort [10]. However, this finding is still consistent with the low incidence of cervical cancer in the EMENA and both measres are nearly equal [26,27]. On the other hand, no statistical differences in the demographic data and HPV DNA positivity between Qatari and non-Qatari Arab women were observed, which is consistent with our previous report [10]. Refelctive of the overall pattern of sexually transmitted infections in EMENA [28,29], it is likely that the infection is transmitted sexually to the infected women from their spouses. Moreover, the rising tourism and the high frequency of expatriates (75%) residing in Qatar [30], hinders drawing inferences about the local dynamics of the infection and prevailing norms of sexual partenring.

In the present study, the high-risk HPV genotypes 16 and 59 were the most common among women with abnormal cytology, while HPV 35, 33, 39, 59 were found among those with normal cytology. HPV 81, 90, 11 were the most prevalent low-risk genotypes among women with both normal and abnormal cytology, a finding that is consistent with our previous study [10]. HPV 81 was also the most frequent LR genotype among women with normal (34.5%) and abnormal cytology (25%), and this also corroborates our previous findings in this population [10].

The findings on HPV DNA prevalence and genotype distribution, as well as HPV seroprevalence and potential risk factors for HPV infection, have the clinical potential to improve cervical cancer screening through identifying women at high risk for cervical dysplasia/cancer. Furthermore, they inform the development of genotype-specific vaccines for trials in population-level programs. It has been shown that HPV serology may underestimate infection exposure, as many women do not develop an HPV antibody response [31]. Though, seroprevalence studies has their limitations, such studies, such as the present study, provide a useful tool to understand the dynamics of HPV infection, thereby providing a baseline assessment for the incorporation of HPV vaccination programs in the State of Qatar. In the present study, we found a lower seroprevalence of 4.5% for HPV-IgG antibodies in Arab women than globally [32,33]. This low seroprevalence supports lower intensity of HPV transmission in the Arab female population, possibly due to poorly connected and sparse sexual networks, a result of the more conservative sexual norms in this part of the world [28].

Interestingly, none of the women were found positive for HPV IgM antibodies and there was no evidence of an association between behavioral risk factors and HPV seropositivity. Furthermore, it has been reported that once seroconversion occurs, anti-HPV antibody levels remain detectable for years [34,35], but in the present study, all the HPV DNA positive women were HPV seronegative. The reason could be that these women never seroconverted, despite acquiring HPV infection previously [34,36]. Additionally, there was no evidence of higher HPV DNA prevalence and seroprevalence among young women in this study, further supporting a flat distribution for HPV prevalence by age. This finding is consistent with our previous findings [10] and those reported in other limited-resource countries in Africa, Asia and globally [32,37]. This finding however contrasts with the common sharp peak in prevalece of HPV infection among young women following their sexual debut in most studies globally [27,38]. Lastly, many potential risk factors have been established for HPV infections but none of these (marital status, education level, economic status, smoking, usage of contraception and awareness of HPV, cervical cancer and HPV vaccine) were associated with HPV DNA positivity or antibody positivity in the present study.

The strengths of the present study lie in the use of a standardized and sensitive molecular assay for HPV detection, rendering our findings amenable to a detailed analysis of HPV prevalence, distribution of HPV genotypes, seroprevalence and risk factors among general population of Arab women residing in the State of Qatar, and comparison to global patterns. In the present study, we aimed for such analysis to be relevant for planning of health service provision and development of appropriate interventions based on the current characteristics of the infection burden and implied future trends for squamous intraepithelial lesions and cervical cancer. The indications that HPV infection burden might be increasing in EMEMNA, adds further importance to this investigation.

The limitations of this study include the fact that, because of socio-cultural context, we were unable to collect detailed data on sexual behaviour such as number of sexual partners, age at first sexual intercourse and extramarital relationships. Additionally, the present study was based on a convenient sample from women attending the Women hospital and Gynecology clinic at PHCC. Therefore, it is not known how representative is this sample of the wider Arab women population residing in the State of Qatar.

## Conclusion

The overall HPV DNA prevalence and seroprevalence among Arab women in the State of Qatar are rather low in comparison to other countries. Despite the low prevalence, there is a diverse distribution of HPV genotypes among Arab women living in Qatar, and there appears to be an increase in prevalence over the last decade. Our study suggests that these genotypes should also included in future vaccines targeting this specific population. Furthermore, the information about the molecular and sero-prevalence of HPV infection will be helpful for policy makers in making an informed decision regarding introduction and development of policies, guidelines and implementation of HPV vaccination in the State of Qatar. Contrary to our expectations, no statistically significant association was observed between HPV DNA positivity or antibody positivity and potential risk factors for HPV infection. Though, Arab women in Qatar reported knowledge of cervical cancer, there was limited knowledge of its link to HPV infection and HPV vaccination. Awareness programs of risk associated with HPV infection and HPV vaccination are warranted in Qatar. Finally, further observational studies of HPV infection levels and HPV incidence among different age groups may help elucidate several poorly understood aspects of HPV epidemiology in this part of the world.

## Supporting Information

S1 Questionnaire Structured questionnaire. (PDF)
