# Supplementary material for: Human Papillomavirus (HPV) Infection: Molecular Epidemiology, Genotyping, Seroprevalence and Associated Risk Factors among Arab Women in Qatar
Source: PLoS One. 2017 Jan 3;12(1):e0169197. doi: 10.1371/journal.pone.0169197 (PMC5207789; doi:10.1371/journal.pone.0169197)
Supplement: S1 Questionnaire — (PDF) [file pone.0169197.s001.pdf]

Bar code: ___________

STUDY QUESTIONNAIRE

Human Papillomavirus in Arab Countries: Molecular, Clinico-Pathological, Serological, and Vaccine Impact Studies in Select Arab Countries

Protocol # 10165/10

Country: ____________________

Date form completed: __ __/__ __/__ __ __ __
DD MM YYYY

Hospital/Health Center/Clinic: ___________________________

Section I. Socio-demographics

1. Nationality: ____________________
2. Age: __ __ years
3. What is your current marital status?
   ☐ Married ☐ Separated/divorced ☐ Widowed
4. Have you been married more than once: ☐ No ☐ Yes **If no, please go to question 6**
5. How many times have you been married: _____ times
6. Please list the date (s) that you were married:
   __ __/__ __ __ __ (MM YYYY) __ __/__ __ __ __ (MM YYYY) __ __/__ __ __ __ (MM YYYY)
7. Does/did your husband / did any of your husbands have other wives: ☐ No ☐ Yes
8. How many children do you have? ☐ 0 ☐ 1 ☐ 2 ☐ 3 ☐ > 3
9. Do you read/write? ☐ No ☐ Yes
10. What is the highest level of school you have completed or the highest degree that you have received?
    ☐ No schooling ☐ Non-college post high school
    ☐ Elementary school ☐ College degree
    ☐ Intermediate school ☐ Undergraduate university degree
    ☐ Secondary/High school ☐ Post-graduate university degree
11. What is your current working status: ☐ Working ☐ Housewife/unemployed
    If housewife or unemployed, please go to question 13
12. What is your occupation: ____________________________________
13. What is your household monthly income?
    ☐ < 5,000 QR ☐ 5,000-10,000 QR ☐ 15,000-20,000 QR ☐ > 20,000 QR ☐ Don’t know
14. How many people including yourself live in your household? _____ people
15. How many rooms (excluding kitchen and bathrooms) does your house have? _____ rooms

Section II. Women’s health

16. What is your height? _____ cm
17. What is your weight? _____ Kg
18. Do you smoke cigarettes? ☐ No ☐ Yes **If no, please go to question 20**
19. How many cigarettes do you smoke per day? _____ cigarettes/day
20. Do you smoke *Sheesha*? ☐ No ☐ Yes **If no, please go to question 22**

21. How many *Sheeshas* do you smoke per week? _____ *sheeshas*/week
22. Are you currently using any method of contraception to prevent pregnancy? ☐ No ☐ Yes

    If no, please go to question 24
23. What type of contraceptives do you use? (Tick all that applies)

    ☐ Birth control pills ☐ Condoms ☐ Intrauterine device ☐ Rhythm method

    ☐ Abstinence (not having sex) ☐ Withdrawal ☐ Female sterilization (tubes tied)

    ☐ Other, specify: ______________________________
24. During the last 12 months, have you had a disease which you got through sexual contact?

    ☐ No ☐ Yes ☐ Don't know
25. During the last 12 months, have you had a bad smelling abnormal genital discharge?

    ☐ No ☐ Yes ☐ Don't know
26. During the last 12 months, have you had a genital sore or ulcer?

    ☐ No ☐ Yes ☐ Don't know

    If the answer is 'no' or 'don't know' to questions 24, 25 and 26, please go to question 28
27. The last time you had any of the problems listed in questions 23-25, did you seek any kind of medical treatment or advice?

    ☐ No ☐ Yes

28. Have you ever had a Pap test (Pap smear)? ☐ No ☐ Yes **If no, please go to question 36**
29. How often do you have a Pap test?

    ☐ Every six months ☐ One a year ☐ Every other year ☐ Rarely
30. Have you ever had an abnormal Pap test? ☐ No ☐ Yes **If no, please go to question 34**
31. How many abnormal Pap tests have you had? _____ tests
32. When was your first abnormal Pap test? ☐ less than a year ago ☐ _____ years ago
33. Since your first abnormal Pap test, have you had a Pap test with a worse result?

    ☐ No ☐ Yes ☐ Don't know
34. When was your most recent Pap test? __ __/__ __ __ __

    MM YYYY
35. What was the result of your most recent Pap test?

    ☐ Normal (negative) ☐ Abnormal ☐ Haven't gotten the results yet
36. Have you heard of HPV? ☐ No ☐ Yes
37. Have you heard of cervical cancer? ☐ No ☐ Yes

    If the answer is 'no' to questions 35 and 36, please go to question 40
38. Have you heard of a vaccine for HPV or cervical cancer? ☐ No ☐ Yes

    If no, please go to question 40
39. Have you gotten the HPV vaccine? ☐ No ☐ Yes

40. Have any of your female family members been diagnosed with cervical cancer? ☐ No ☐ Yes

    If no, please go to question 42
41. What is their relationship to you?

    Person 1: ________________ Person 2: ________________ Person 3: ________________

THANK YOU FOR YOUR PARTICIPATION
